# Cervical cancer with Human Papilloma Virus and Epstein Barr Virus positive

**DOI:** 10.1186/1477-3163-5-13

**Published:** 2006-05-10

**Authors:** Adi Prayitno

**Affiliations:** 1 Department of Pathology Anatomic, Faculty of Medicine, University of Sebelas Maret, Surakarta, Indonesia

## Abstract

The Early-7 (E7) protein of HPV binds to the underphosphorelated form of the tumor suppressor protein – pRb and displaces the E2F transcription factor that is normally bound by pRb. The latent membrane protein-1 (LMP-1) of EBV prevents apoptosis of B cells by up regulating the expression of bcl-2, and it activates growth promoting pathway that are normally triggered by T cell – derivate signal. The aims of this study to know that in cervical cancer stay HPV and EBV.

DNA was isolated from nineteen sample cervical cancer tissues frozen section. Diagnose related with HPV and EBV was made by Polymerase Chains Reaction (PCR).

The result of this experiment showed that from 19 samples diagnosed as cervical cancer, 17 samples are positive HPV and 13 samples had HPV and EBV positive. The conclusion of this experiment is 89% of cervical cancers are infected with HPV and 68% also infected with HPV and EBV.

## Introduction

The traditional way to diagnose tumors is by histopathology stains and analysis. The diagnosis of cancer relies primarily on invasive tissue biopsy [1]. The conventional histopathology based on light microscopy, however, has recently been complemented with ultrastructure, immunohistochemistry and molecular diagnostics [2].

Cancer of the uterine cervix is the most common form of cancer in women developing countries as leading cause of cancer-related deaths in women in the world as a whole [3]. Cervical cancer is stay as the first for cancer caused death in Indonesia and still be a problem of health [4]. There are four human viruses that cause cancer in human. There are papilloma viruses (PV), epstein barr viruses (EBV), hepatitis B virus (HBV), and kaposi sarcoma herpes virus (KSHV) [3].

The E7 protein binds to the underphosphorelated form of the tumor suppressor protein pRb and displaces the E2F transcription factor that is normally bound by pRb [3,5,6].

The latent membrane protein-1 (LMP-1) of EBV prevents apoptosis of B cells by up regulating the expression of bcl-2, and it activates growth promoting pathway that are normally triggered by T cell-derivated signal [3,5,6].

## Materials and methods

Nineteen frozen section tissues are collected from obstetric and gynecologic part of doctor Muwardi Hospital Surakarta patient with cervical cancer from January 2001 to January 2002. DNA isolation was made by Henk Schmits method with some modifications. Cut up to 25 mgr of tissue into small pieces, place in 1.5 ml a microfuge tube volume, and add 200 ul of DNA extraction buffer. Add 20 ul of Proteinase K stock solution, mix by vortexing, and incubate at 55°C overnight [7].

Diagnose related with HPV infections are made by Henk Schmits and/or Nigel McMillan and Nina Fowler PCR-method with some modifications [7,8]. Diagnose related with EBV infections are made by Ausbel PCR-method with some modifications [9]. Twenty five μl microfuge tube Ready To Go PCR Bead (Amersham Pharmacia Biotech) mixed with 2 μl HPV consensus primers (MY09 and MY11) (CYBERGENE AB) and 2 μl DNA template the other hand twenty five μl microfuge tube Ready To Go PCR Bead (Amersham Pharmacia Biotech) mixed with 2 μl Ebna 3C^5^/Ebna 3C^3 ^primers (CYBERGENE AB). PCR protocol for both amplifications are 94°C for 50 seconds, 59°C for 50 seconds, 72°C for 50 seconds and 4°C soak. The Amplification of HPV-L-1 gene produced 450 bp long. The amplified EBV-Ebna 3C product is 153 bp long as EBV_1 _and 246 bp as EBV_2_.

## Result and discussion

The traditional way of classifying tumors is by histopathology; the staining and analysis of tissue samples. Now, the ability to analyze change in the levels of the transcripts and/or protein products for literally thousands of genes promises interesting possibilities as a research tool – for understanding the underlying molecular mechanisms, but also for automated tissue diagnosis [1,10]. The diagnosis of cancer relies primarily on invasive tissue biopsy, as non invasive diagnostic test are generally insufficient to define a disease process of cancer. Molecular medicine has led to the discovery and application of molecular tumor markers, which make histology more accurate and additionally help to prognosticate cancer. The diagnosis of cancer involves the analysis of tissue and cytology specimens obtained through several procedures, including surgical biopsy, endoscopic biopsy, etc. Polymerase Chain Reaction Method is the technique that based on detection of specific sequences of gene targets by use the specific primer [2,11,12].

The result of this experiment is from 19 cervical cancer samples found 17 samples (89%) with HPV positive.

Neoplasm of the cervical uterine is the most common form neoplasm in women [3,13-20]. This neoplasm is found in developing state as leading cause of neoplasm-related deaths in women in the world as a whole. The mortality rate has been estimated at 2.8 deaths per 100.000 women, with a total of 252 deaths reported in 1993. Now the pathogenesis of cervical cancer is pointed to HPV. Human papilloma virus infection associated with malignancies of urogenital tract and anus. He also thought to be related to disorder of skin and the upper-respiratory tract. Many studies suggest that cervical cancer is caused by sexual transmitted agents, and HPV is a prime suspect [3].

More than 85% of invasive squamous cell cancer infected by HPV types 16 and 18 and, less commonly, types 31, 33, 35, and 51. The affinity of these viral proteins for the products of tumor suppressor genes differs depending on the oncogenic potential of HPV. Early-6 and E7 proteins derived from high-risk HPV bind to pRb and p53 with high affinity. The Early-6 protein of HPV binds to and facilitates the degradation of the p53 gene product. The Early-7 protein binds to the underphosphorelated form of the tumor suppressor protein pRb and displaces the E2F transcription factor that are normally bound by pRb [3,5,6].

Human papilloma viruses as an oncogenic virus are well characterized viruses that play role in cervical cancer. However there are possible that other viruses might also involve in this cancer incidence [3,13-21].

Other result of this experiment is from 17 samples with HPV positive found 13 samples had also EBV positive.

Epstein-Barr viruses known involve in many malignancy cases such as nasopharyngeal carcinoma, limphoma maligna, Burkitts limphoma, Hodgkins disease and gastric carcinoma. It is known that EBV also infected squamous cells [3,13,22-25].

There is increasing evidence that among the concequences of viral infection are influence on cell survival which result from inference, by viral elements, with the normal regulation of apoptosis. Epstein-Barr virus enhancing the survival of B cells via the number of mechanism. Of these, conversion of cell from bcl-2 negative to bcl-2 positive through to transactivating ability of the latent EBV gene LMP-1 and EBNA-2, provides the virus with a strategy for survival during the germinal centre stage of B cell development when expression of bcl-2 protein is normally down regulated [3,5].

From this experiment we found 13 samples (68%) had HPV and EBV positive. Our conclusion is expected HPV may be EBV also involve in carcinogenesis of cervical cancer.

**Figure 1:**
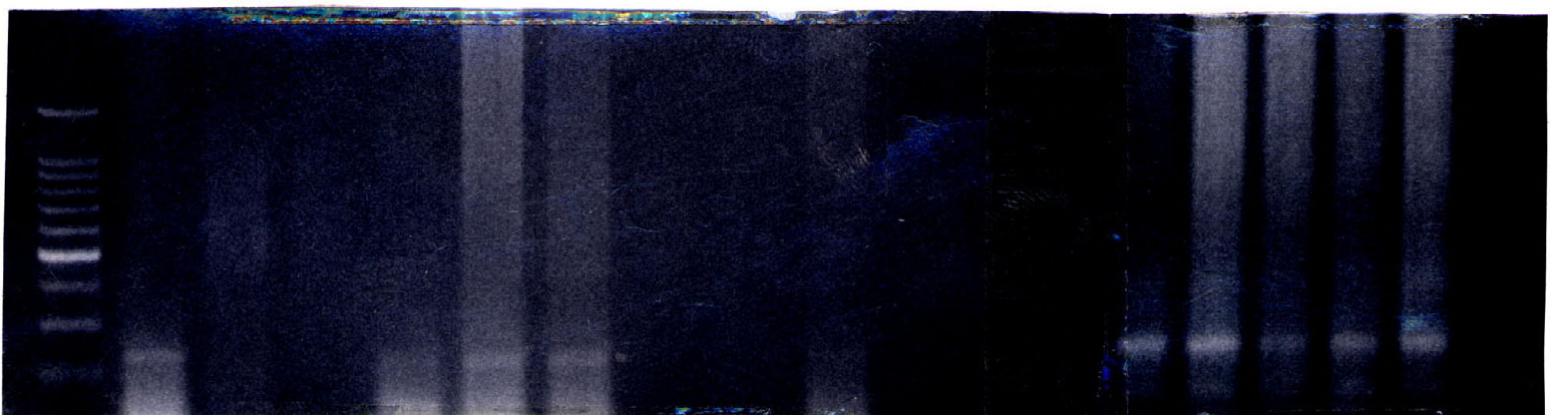
The gel electrophoresis from PCR product to amplified of L-1 gene (450 bps long) of HPV in cervical cancer samples.

**Figure 2:**
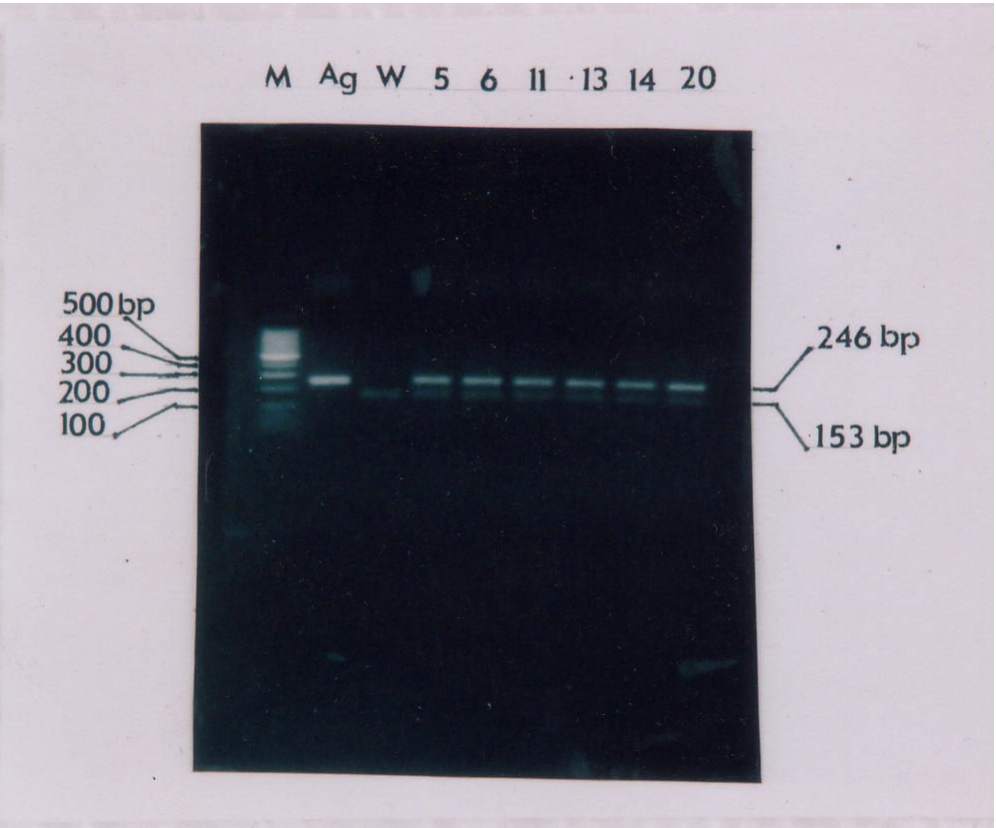
The gel electrophoresis from PCR product to amplified of EBV_1 _(246 bps long) and and EBV_2 _(153 bps long) of EBV in cervical cancer with HPV positive samples.
